# Supplementary material for: Charge Modification as a Mechanism for Tunable Properties in Polymer–Surfactant Complexes
Source: Polymers (Basel). 2021 Aug 20;13(16):2800. doi: 10.3390/polym13162800 (PMC8397960; doi:10.3390/polym13162800)
Supplement: Supplementary file 1 [file polymers-13-02800-s001.zip › polymers-1317212-supplementary.pdf]

Charge modification as a mechanism for tunable properties in polymer/ surfactant complexes

Christopher Hill,† Wasiu Abdullahi,† Robert M. Dalgliesh,‡ Martin Crossman,¶ and Peter C. Griffiths*,†

†*School of Science, Faculty of Engineering and Science, University of Greenwich, Chatham Martitime, Kent, ME4 4TB, UK*

‡*ISIS-CCLRC, Rutherford Appleton Laboratory, Chilton, Oxon, OX11 0QX, U.K*

¶*Unilever Research, Port Sunlight, Quarry Road East, Bebington, Wirral, CH63 3JW, UK*

E-mail: P.Griffiths@greenwich.ac.uk

Supplemental Information

S1. Small-angle neutron scattering (SANS) additional modelling properties

Below in Table are the additional parameters obtained by fitting t he S ANS d ata f or the series of cat-HEC polymers.

Table S1: Fitted structural parameters of the family of cat-HEC polymers. R is radius, L is length and f is fractal dimension.

Degree of modification/ (%)	Background	R/ (Å)	L/ (Å)	ϕ_{fitted}	$\phi_{\text{Calculated}}$
0	0.01	6.6	356	0.003	0.006
0.5	0.03	7.5	109	0.006	0.006
0.95	0.01	7.0	80	0.005	0.006
1.8	0.01	7.9	73	0.003	0.006
2.7	0.02	8.4	78	0.006	0.006

S2. Pulsed gradient spin echo (PGSE) NMR

Method

Experiments were carried out at 25 (± 0.5) °C on a 400 MHz Bruker FT NMR spectrometer. In the PGSE-NMR experiment, a stimulated echo sequence was used, in which the diffusion time (Δ) was set to 600 ms, the duration of the gradient pulses (δ) was held constant at 2 ms and intensity (G) varied from 5 to 950 G cm^{-1}. 32 scans were accumulated over 32 gradient steps and self-diffusion coefficients were extracted by fitting the entire data set via CORE modelling,[1,2] discussed below.

Data analysis

In a PGSE-NMR experiment, the NMR spectrum is recorded as a function of increasing field gradient strength. The fundamental information is contained in the rate of decay of the chemically distinct peaks. The CORE method provides the original dataset in a 3D format with the spectrum running left to right, and increasing field gradient intensity increasing front to back (see Figure S1a for example).

In the CORE analysis, it is possible to "mask" or edit out specific peaks or regions of the spectrum, and this is routinely carried out for the water peak. The CORE analysis then seeks a global minimum for the entire dataset, given a set of input criteria, via a measure of the signal-to-noise, the number of components present (and hence, the number of expected diffusion coefficients) that best describe the attenuation of all of the points in each of the peaks.[3] A significant output from this analysis is the residuals map, being the difference between the fit to (or simulation of) the data, and the original dataset. An appropriate fit in this representation, would be manifest as a small absolute magnitude of noise, and an absence of any coherent waves or significant intensity across regions of the 3D map (see Figure S1c for example).

Masking different regions within the NMR spectrum using the CORE method also allows

for specific components to be analysed separately, i.e. polymer and surfactant diffusion, this being facilitated in mixtures that do not contain overlapping peaks within the NMR spectrum, vis Figure S2. Examples of this modelling approach are shown in Figures S3 and S4, whereby the two components within the mixture containing uncharged HEC polymer + 4 mM SDS are modelled separately. For the polymer specific peaks (Figure S3), it was found that a single stretched exponential was adequate to model the data (no coherent wave in the residual plot, see Figure S3c). Use of a stretched exponential provides a simple way of representing a range of molecular weight. For the SDS peaks (Figure S4), a simple single exponential was found to provide a very good representation of those attenuation functions and therefore describes the data very well.

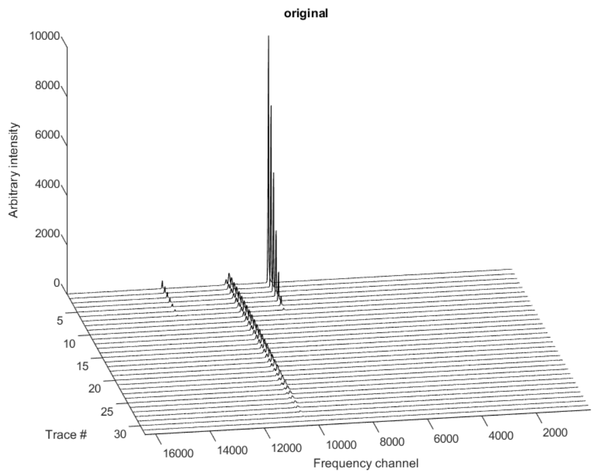

(a) Original data

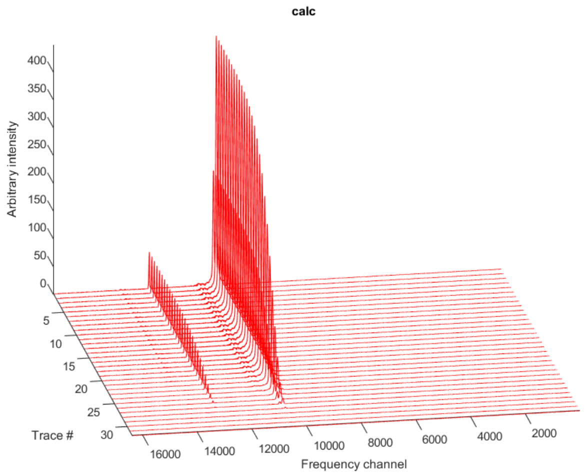

(b) Fit of the polymer peaks, solvent masked, single stretched (polydispersed) exponential

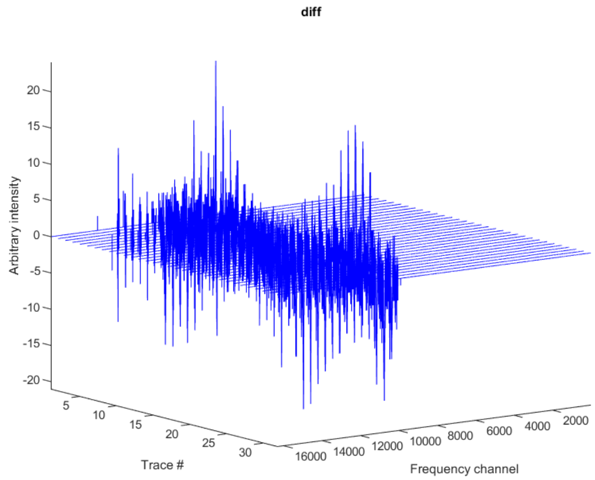

(c) Residual plot

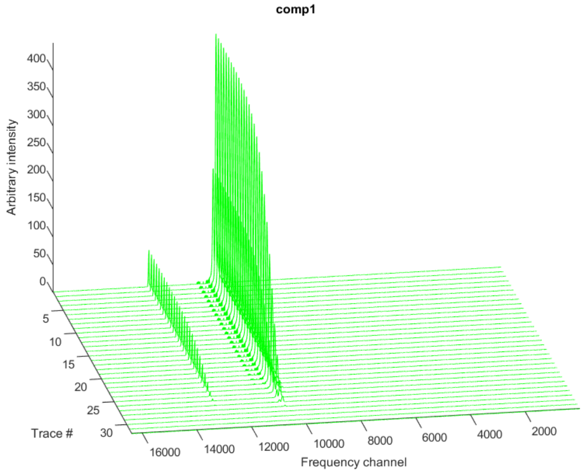

(d) Polymer diffusion

Figure S1: CORE analysis of an aqueous solution of 1 wt% N = 0% uncharged cat-HEC polymer. Here the solvent (D_2O) has been masked in the analysis, hence the fitting routine has been optimised for the polymer peaks. Note the relative y-scales.

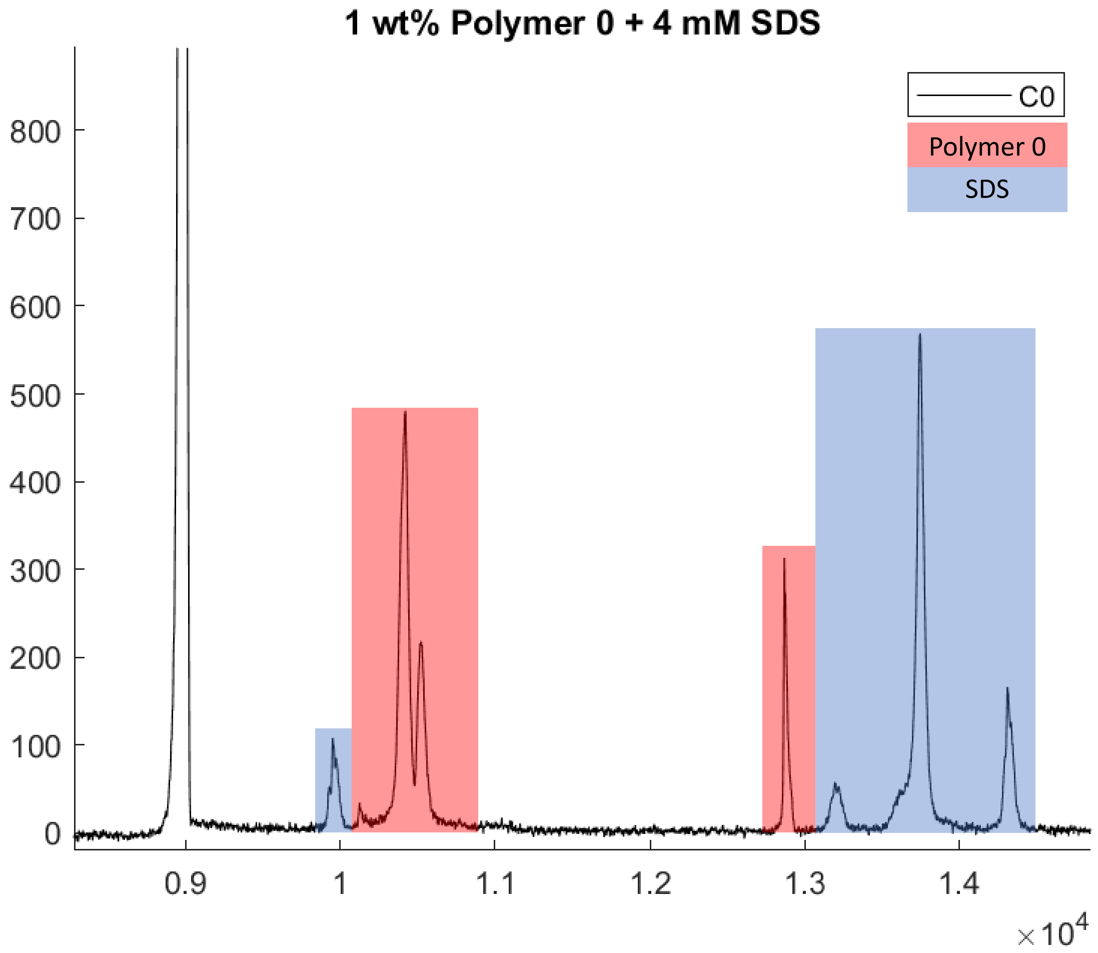

Figure S2: 1D NMR spectrum of 1 wt% Polymer 0 + 4 mM SDS. Polymer and surfactant regions highlighted separately to show different regions of analysis: red = polymer peaks, blue = SDS, white peak at the 0.9 x 10^4 frequency channel is water.

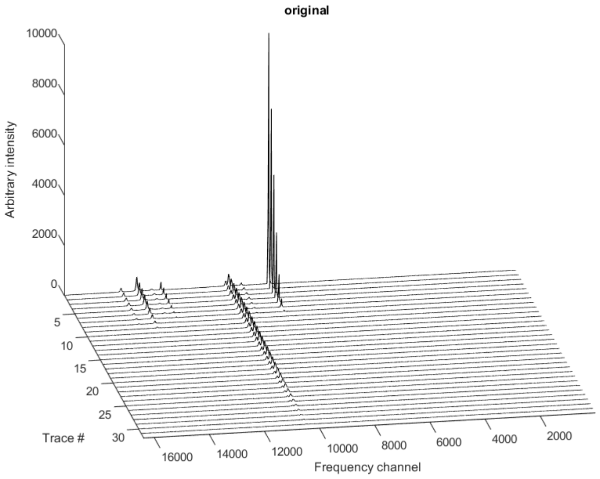

(a) Original data

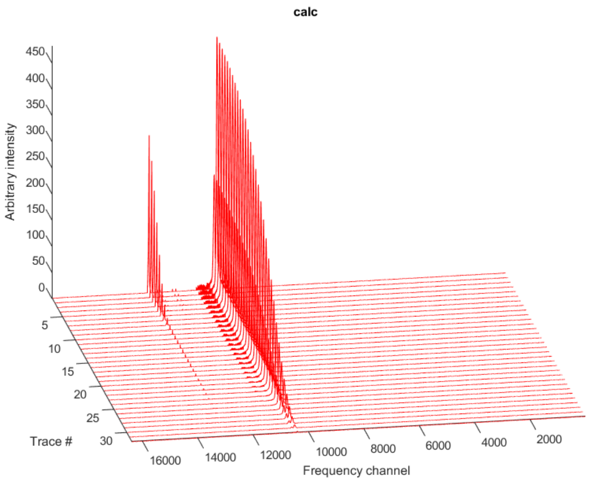

(b) Fit of the polymer peaks, solvent/ SDS masked, single stretched (polydispersed) exponential

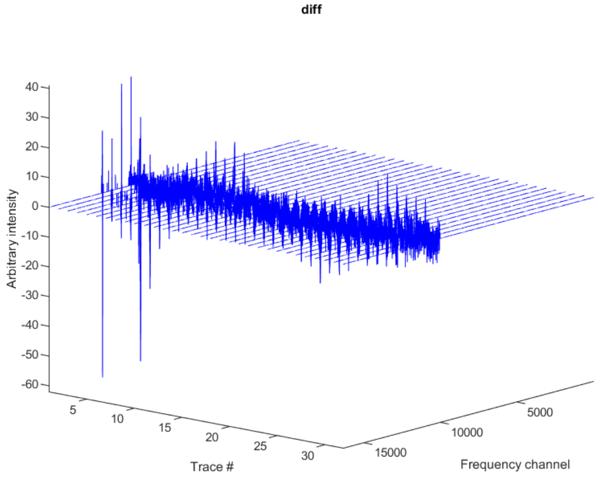

(c) Residual plot

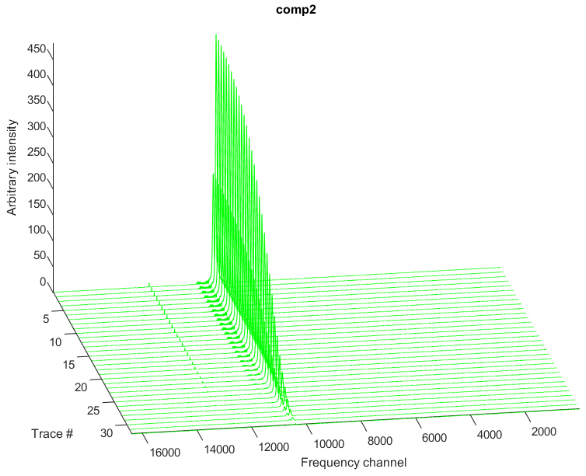

(d) Polymer diffusion

Figure S3: CORE analysis of an aqueous solution of 1 wt% N = 0% uncharged cat-HEC polymer + 4 mM SDS. Here the solvent (D_2O) and SDS has been masked in the analysis, hence the fitting routine has been optimised for the polymer peaks. Note the relative y-scales.

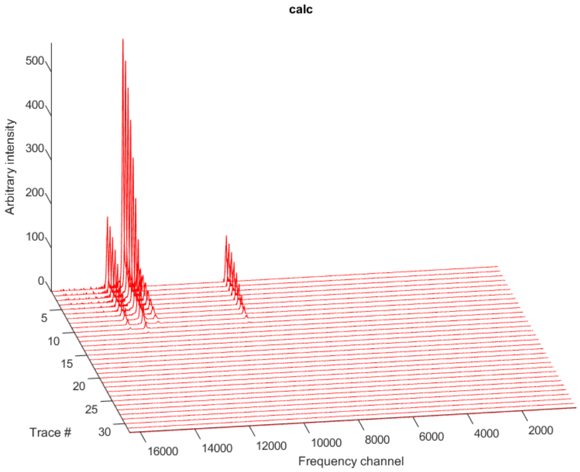

(a) Fit of the SDS peaks, solvent/ polymer masked, single exponential

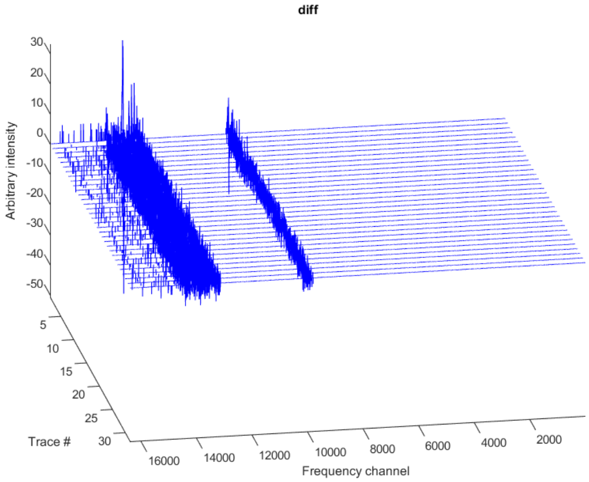

(b) Residuals

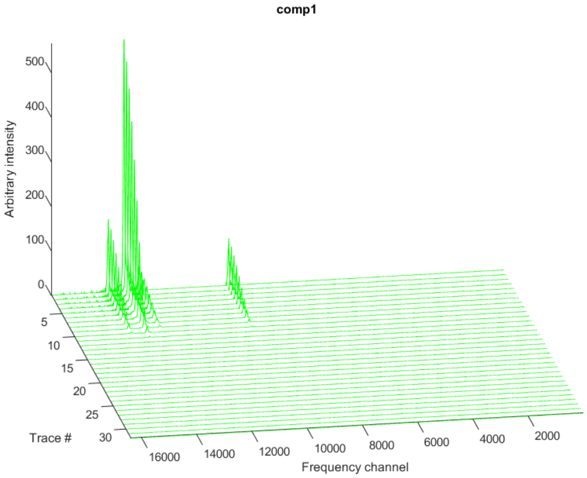

(c) SDS diffusion

Figure S4: CORE analysis of an aqueous solution of 1 wt% N = 0% uncharged cat-HEC polymer + 4 mM SDS. Here the solvent (D_2O) and polymer has been masked in the analysis, hence the fitting routine has been optimised for the SDS peaks. Note the relative y-scales.

Below in Tables 2 and 3 are the parameters obtained by fitting the PGSE NMR data using the CORE approach for the uncharged HEC polymer + SDS mixtures. As mentioned above, the CORE approach allows for the different components within the sample to be analysed separately through masking of specific c omponents w ithin t he N MR s pectrum, h ence the Tables below present the data for the polymer and SDS diffusion separately.

These data echo previous results reported by Patel *et al.*[3] as well provide additional evidence within the main text to demonstrate the lack of significant i nteraction between the uncharged HEC polymer and SDS. Direct comparison of both sets of data show that there is little to no change in the measured diffusion coefficient of the polymer or surfactant when present in the mixture, implying minimal or transient interactions between these two components.

Table S2: Parameters obtained by fitting PGSE NMR data using the CORE approach. Here the surfactant (SDS) is masked in the analysis and the parameters therefore represent diffusion coefficients obtained for the polymer only. D_s is the polymer self-diffusion coefficient, R_h is the calculated hydrodynamic radius and β is the polydispersity of the polymer component.

Sample Description	D_s/ 10^{-11}(m^2/s)	R_h/ (nm)	Temp/ K
Polymer 0	0.13 β=0.47	190	298.5
Polymer 0 + 2 mM SDS	0.15 β=0.50	170	298.0
Polymer 0 + 4 mM SDS	0.15 β=0.52	170	298.0

Table S3: Parameters obtained by fitting PGSE NMR data using the CORE approach. Here the polymer is masked in the analysis and the parameters therefore represent diffusion coefficients obtained for the surfactant only. D_s is SDS self-diffusion coefficient, R_h is the calculated hydrodynamic radius. Note $C_{SDS} < $ CMC

Sample Description	D_s/ 10^{-10}(m^2/s)	R_h/ (nm)	Temp/ K
SDS	4.5	0.5	298.0
Polymer 0 + 2 mM SDS	4.5	0.5	299.0
Polymer 0 + 4 mM SDS	4.5	0.5	298.0

References

(1) Stilbs, P.; Paulsen, K.; Griffiths, P. Global least-squares analysis of large, correlated spectral data sets: application to component-resolved FT-PGSE NMR spectroscopy. *The Journal of Physical Chemistry* **1996**, *100*, 8180–8189.

(2) Persson, K.; Griffiths, P.; Stilbs, P. Self-diffusion coefficient distributions in solutions containing hydrophobically modified water-soluble polymers and surfactants. *Polymer* **1996**, *37*, 253–261.

(3) Patel, L.; Mansour, O.; Crossman, M.; Griffiths, P. Electrophoretic NMR Characterization of Charged Side Chain Cationic Polyelectrolytes and Their Interaction with the Anionic Surfactant, Sodium Dodecyl Sulfate. *Langmuir* **2019**, *35*, 9233–9238.
